# Comparative Pharmacokinetic Studies of Four Ginsenosides in Rat Plasma by UPLC-MS/MS after Oral Administration of *Panax quinquefolius*-*Acorus gramineus* and *Panax quinquefolius* Extracts

**DOI:** 10.1155/2019/4972816

**Published:** 2019-11-06

**Authors:** Hailong Xie, Dongxue Wang, Wenjun Zhang, Xinjia Yan, Ying Zhao

**Affiliations:** ^1^College of Pharmacy, Heilongjiang University of Chinese Medicine, 24 Heping Road, Harbin 150040, China; ^2^College of Pharmacy, Harbin University of Commerce, 138 Tongda Road, Harbin 150076, China

## Abstract

*Panax quinquefolius* (PQ) and *Acorus gramineus* (AG) are drug target pairs in traditional Chinese medicine (TCM), which are used to treat age-related diseases. In the present study, we simultaneously determined the contents of four main bioactive ginsenosides (Rb_1_, Rb_2_, Rd, and Re) in rat plasma using an ultrahigh-performance liquid chromatography-tandem mass spectrometry (UPLC-MS/MS) method. Plasma specimens were purified by using the solid-phase extraction procedure, and separation was performed on Waters ACQUITY UPLC BEH C_18_ (100 mm × 2.1 mm, 1.7 *μ*m) in multiple reaction monitoring (MRM) mode and negative electrospray ionization (ESI) mode. The established UPLC-MS/MS method showed good linear correlation (*r* ≥ 0.9978), stability (−11.93 to 12.11%), precision (RSD < 14.63%), and recovery (76.43%–95.20%). The lower limit of quantification was 3.6 ng/mL for Rb_1_, 1.6 ng/mL for Rb_2_, 1.2 ng/mL for Rd, and 2.5 ng/mL for Re. This validated method was successfully employed to investigate the pharmacokinetics of the four ginsenosides in rat plasma after oral administration of PQ-AG and PQ extracts. The results revealed the pharmacokinetic profiles of PQ-AG drug pair and clarified that AG played a critical role in stimulating the absorption of active ginsenosides in PQ. Collectively, our findings provided valid and reliable evidence for the rational use of PQ-AG in clinical practice.

## 1. Introduction

In multiple diseases caused by complex pathogenic factors, the advantages of using a single herb are absent or show weaker meaning. The drug target pairs are the combination of two relatively fixed drugs based on the theory of traditional Chinese medicine (TCM), showing important clinical significance in various diseases [1–4]. *Panax quinquefolius* (PQ) is used to restore vital energy and treat diabetes [5] and tumor [6] as Qi-tonifying agents in TCM. *Acorus gramineus* (AG) possesses the effects of stimulating appetite [7], resolving dampness [8], inducing resuscitation [9], improving learning and memory [10], and so on. Moreover, AG can promote drug absorption [11, 12]. As described in early publications and our previous studies, PQ-AG drug pair is popular due to its remarkable and reliable therapeutic actions, such as antioxidant, antiaging, and blood circulation promoting properties, especially in treating diabetes [13–15].

A great progress has been made in pharmacological research of ginsenosides. However, only few pharmacokinetic studies have investigated drug pairs. In recent years, some methods have been used for the determination of ginsenosides. An LC-MS/MS method has been established to determine the concentrations of ginsenosides in plasma and brain [16]. Moreover, an HPLC-MS/MS method has been used to characterize and quantify ginsenosides in plant extracts from *Panax ginseng* and PQ [17]. An LC-MS fingerprint and a chemometric approach, in combination with multivariate statistics, have been used to distinguish between *P*. *quinquefolius* samples derived from the United States and China [18].

**Figure 1:**
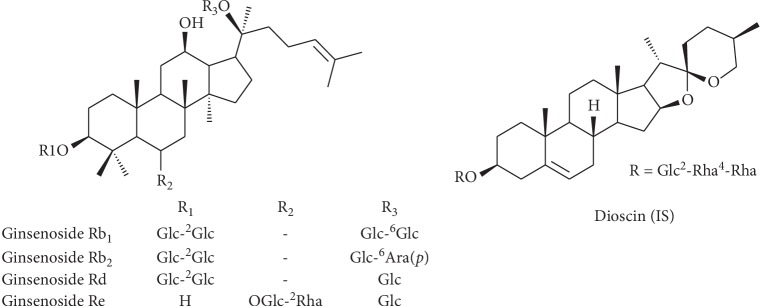
The chemical structures of Rb_1_, Rb_2_, Rd, Re, and dioscin (IS).

Because only few reports have assessed the pharmacokinetic profiles and the compatibility mechanism of the drug pair, we developed an ultra-performance liquid chromatography coupled with a triple quadrupole electrospray tandem mass spectrometry (UPLC-MS/MS) method to compare the pharmacokinetic profiles of four major bioactive components of ginsenosides, Rb_1_, Rb_2_, Rd, and Re (Figure 1) in rat plasma after oral administration of PQ-AG and single PQ extracts. Collectively, our findings provided scientific evidence on reasonable compatibility of the drug pair and laid the foundations for further investigation of the behavioral mechanism.

## 2. Materials and Methods

### 2.1. Chemicals and Reagents

Rb_1_ (MUST-14032301), Rb_2_ (MUST-14072210), Rd (MUST-16012503), Re (MUST-14091710), and dioscin (MUST-15012101) (IS, purity ≥ 98%) were purchased from Chengdu MANSITE Pharmaceutical Co., Ltd. (Chengdu, Sichuan, China).

PQ and AG were collected from Heilongjiang Ruixiang Pharmaceuticals Company (Harbin, Heilongjiang, China) and identified by Ruifeng Fan from the Heilongjiang University of Chinese Medicine. The plant species were deposited (specimen 2015093104) at the Pharmacy College. Acetonitrile and methanol were of HPLC grade and purchased from Fisher (Emerson, America). Formic acid was of HPLC grade and purchased from Sigma-Aldrich (St Louis, MO, USA). All other reagents were of analytical grade.

### 2.2. Animals

Male SD rats (230 ± 20 g) were obtained from the Laboratory Animal Center of Changchun Yisi (Changchun, Jilin, China). The animal experiments were performed in accordance with the Guide for the Care National Institutes of Health. Before the experiments, the rats were fed in an environmentally controlled room with temperature of 22 ± 2°C, humidity of 50 ± 10%, and 12 h dark-light cycle for 5 days.

### 2.3. Chromatographic Conditions

Chromatographic analysis was performed on a Waters Acquity UPLC system (Waters Corp., Milford, MA, USA). An ACQUITY UPLC BEH C_18_ (100 × 2.1 mm, 1.7 *μ*m) column was used for all analytes. The column temperature was at 35°C. The mobile phase was composed of formic acid aqueous solution (A, 0.1%) and acetonitrile (B) at a gradient elution of 25% B at 0–0.5 min, 25%–50% B at 0.5–2.0 min, 50–60% B at 2.0–5.0 min, 60–25% B at 5.0–5.5 min, and 25% B at 5.5–7.0 min, with a flow rate of 0.2 mL/min.

### 2.4. MS Conditions

**Figure 2:**
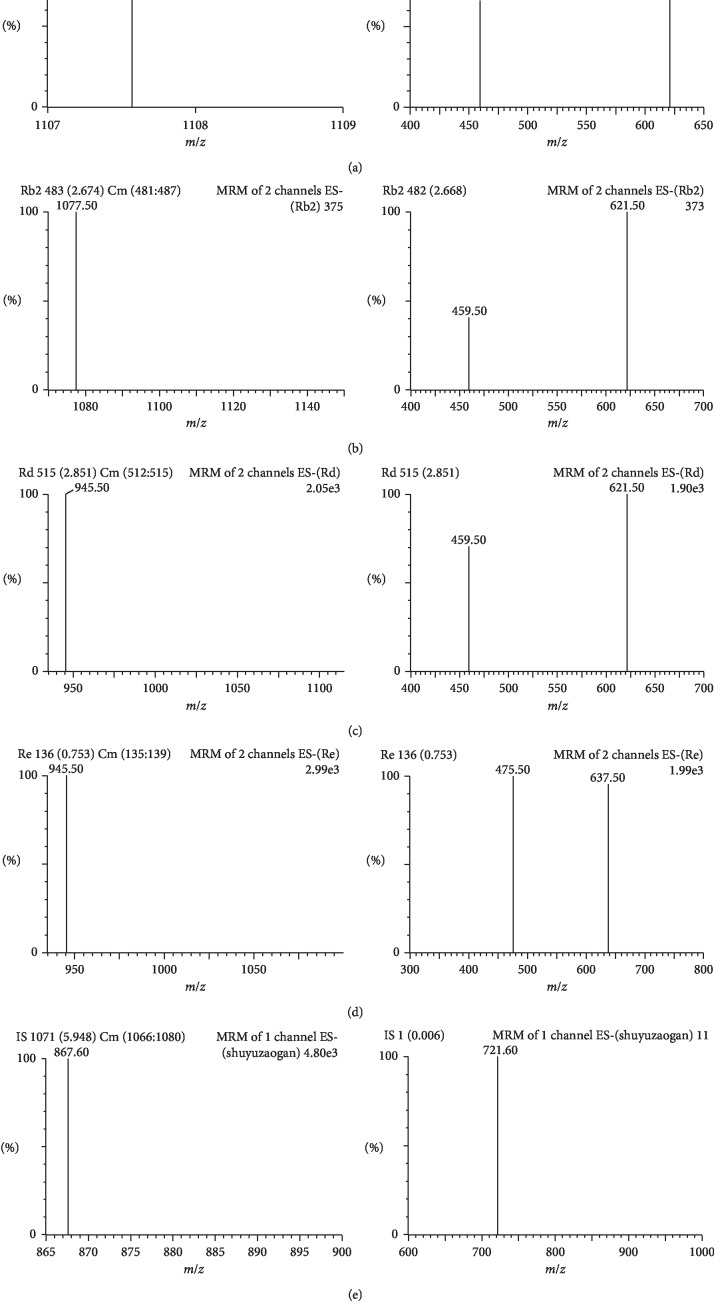
Product ion mass spectra of Rb_1_ (a), Rb_2_ (b), Rd (c), Re (d), and dioscin (e).

MS detection was performed using the Xevo Triple Quadruple MS (Waters Corp.) equipped with an ESI source in both positive and negative ionization modes. The experimental parameters were set as follows: CV 2.80 kV; desolvation temperature 350°C; source temperature 650°C; cone gas flow 50 L/h; and desolvation gas flow 650 L/h. The detection was carried out using the MRM scan mode. The CV and CE were optimized for each analyte, and selected values are listed in Table 1 and Figure 2. All experimental data were collected in centroid mode using Masslynx 4.1 software (Waters Corp., Milford, USA), and postacquisition quantitative analysis was conducted using Target Lynx program (Waters Corp., Milford, USA).

### 2.5. Preparation of PQ-AG and PQ Extracts

The powder of PQ (0.5 kg) and PQ-AG (1.0 kg, with equal proportions of PQ and AG) was extracted for three times by refluxing with water (1 : 10, w/v) for 2 h of each time. Then, the extracts were filtered, and the filtrates were pooled and concentrated to dryness under reduced pressure by rotary evaporator.

### 2.6. Preparation of Calibration Standards and QC Samples

The standard stock solution was prepared in methanol with four ginsenosides at the final concentration of 1.14 mg/mL for Rb_1_, 0.10 mg/mL for Rb_2_, 0.78 mg/mL for Rd, and 1.58 mg/mL for Re. The working standard solution at required concentration was obtained by continuously diluting the stock solution. The IS stock solution was also prepared with methanol at a final concentration of 720 ng/mL. Calibration samples were prepared by mixing solutions of standard mixture, internal standard, and methanol with rat blank plasma at the final concentrations of 3.6, 7.2, 35.6, 71.2, 142.4, 284.8, 569.5, and 1, 139.0 ng/mL for Rb_1_, 1.6, 3.2, 6.3, 31.3, 62.5, 125.0, 250.0, and 500.0 ng/mL for Rb_2_, 1.2, 2.4, 4.9, 24.4, 48.8, 97.6, 195.1, and 390.2 ng/mL for Rd, and 2.5, 4.9, 9.9, 49.3, 98.6, 197.1, 394.3, and 788.5 ng/mL for Re. QC samples were also prepared with the same method at low, middle, and high concentrations (7.2, 142.4, and 911.4 ng/mL for Rb_1_, 3.2, 62.5, and 400.0 ng/mL for Rb_2_, 2.4, 48.8, and 312.2 ng/mL for Rd, 5.0, 98.6, and 630.8 ng/mL for Re). All solutions were stored at 4°C.

### 2.7. Preparation of Plasma Samples

Plasma specimens (200 *μ*L) were mixed with IS solution (20 *μ*L, 720 ng/mL) in an Eppendorf tube and centrifuged at 4, 000 rpm for 15 min at 4°C. Then, the supernatant was removed from plasma sample. Hydrochloric acid (4 *μ*L) was added into 200 *μ*L plasma sample, and then, the mixture was vortexed for 1 min. Waters OASIS HLB SPE C18 columns (Waters, USA) were used to remove the proteins in plasma samples, and it was preactivated by 3 mL methanol and 3 mL water. Then, 2 mL of 100% methanol was used to wash the column, and the eluate was collected and dried under nitrogen gas (45°C). Subsequently, 100 *μ*L of 100% methanol was used to redissolve the residues, followed by centrifugation at 13, 000 rpm for 15 min at 4°C. Before UPLC-MS/MS analysis, the sample was finally filtered through a 0.22 *μ*m membrane filter.

### 2.8. Method Validation

The selectivity, linearity, precision, accuracy, extraction recover, matrix effects (ME), and stability were determined based on the FDA guidelines [19]. To verify whether there was endogenous interference, the specificity between the blank plasma and plasma samples after oral administration of PQ and PQ-AG extracts was evaluated by chromatographic comparisons. The precision and accuracy of analytical methods were expressed as %CV and the relative standard deviation (RSD) value for the QC samples. Recoveries were determined by the peak areas of the standard plasma and pure reference samples. The stability assays were carried out to demonstrate the experimental conditions of the samples.

#### 2.8.1. Selectivity

Chromatograms of six different blank plasma samples were compared with those of the correspondingly spiked plasma and the plasma samples after oral administration of PQ-AG and PQ extracts to assess the selectivity.

#### 2.8.2. Linearity and LLOQ

The calibration curves were constructed by plotting the peak area ratio versus the concentrations of the four analytes with a weighted (1/*x*^2^) least square linear regression using spiked plasma samples at six concentrations. The LLOQ was defined as the lowest analytical concentration of the calibration curve, at which the measured precision expressed as RSD was within ±20% and the accuracy was within ±20%.

#### 2.8.3. Precision and Accuracy

The intraday and interday precisions as well as accuracies were evaluated by determining the ginsenoside concentration in QC samples at three different levels (LQC, MQC, and HQC) in six replicates on the same day and during three different days. The precision was defined as RSD of the measured concentration, and the accuracy (%) of the measured mean value was deviated from the nominal value. The intraday and interday precisions were within 15%. The accuracy was within 80∼120%. The RSD of LLOQ samples should be within 20%.

#### 2.8.4. Extraction Recovery and ME

Blank plasma samples from six rats were extracted and then spiked with analytes and IS to evaluate the ME. ME was evaluated by comparing the peak areas of QC samples at three concentration levels after sample preparation with those prepared in solution at the same concentration level. Recovery was calculated by comparing the peak areas of QC samples with those spiked after sample preparation at the same concentration level.

#### 2.8.5. Stability

The stability of the four analytes was determined by exposing plasma samples to QC at three concentration levels in six replicates. Samples were stored at −80°C for 1 month to assess the short-term storage stability. Freeze-thaw stability was performed after three freeze-thaw cycles at −80°C during three consecutive days. To evaluate the postpreparative stability, extracted QC samples were kept in the autosampler (4°C) for 12 h before analysis. The stability assessment of these samples was calibrated with freshly prepared standard as previously described.

### 2.9. Statistical Analysis

DAS 3.2 software (Mathematical Pharmacology Professional Committee of China, Shanghai, China, 2011) was applied for the analysis of concentration-time data to investigate the pharmacokinetic parameters of four ginsenosides in various groups. Data were presented as the mean ± SD (standard deviation) with triplicate measurements. A significance level was set at *P* < 0.05.

### 2.10. Pharmacokinetic Studies

Male SD rats were randomly and evenly divided into two groups (*n* = 6 per group) in pharmacokinetic studies. To evaluate the compatible effect of AG upon PQ treatment, rats were given PQ at a dosage of 0.54 g/kg (equivalent to the amount of raw drug 0.54 g) in the PQ group. PQ-AG was combined with an equal proportion to prepare the mixed extract. The oral dosage in rats was 1.08 g/kg (equivalent to the amount of the raw drug including 0.54 g AG and 0.54 g PQ) in the PQ-AG group. Blood samples were collected at specific time points of 0, 0.25, 0.5, 1.0, 1.5, 2.0, 4.0, 6.0, 8.0, 12.0, 16.0, 24.0, and 48.0 h. All the plasma samples were obtained by centrifuging the blood samples at 3, 500 rpm for 10 min, then labeled and frozen at −80°C prior to analysis.

## 3. Results and Discussion

### 3.1. Optimization of UPLC-MS Conditions

In the optimization process, we found that acetonitrile/H_2_O system offered the best performance among different mobile phases, including methanol/water, acetonitrile/water, and methanol/acetonitrile/water. Formic acid in water could provide the best peak shape among different modifiers. We also optimized additional UPLC conditions, such as column temperature (25, 30, 35, and 40°C) and flow rate (0.2, 0.3, and 0.4 mL/min). The highest selectivity and resolution were obtained for all tested compounds within 7.0 min under the optimized UPLC conditions as follows: Waters ACQUITY UPLC BEH C_18_ (100 × 2.1 mm, 1.7 ìm) column at 35°C and 0.1% formic acid in water (A)-acetonitrile (B) mobile phase at a flow rate of 0.2 mL/min. The precursor ion was [M-H]^−^ at *m*/*z* 1, 107.5 for Rb_1_, and the product ion peak was at *m*/*z* 459.6, which correspond to the loss of four molecules of Glc [M-4Glc-H]^−^. The precursor ion was [M-H]^−^ at *m*/*z* 1, 077.7 for Rb_2_, and the product ion peak was at *m*/*z* 459.5, which correspond to the loss of three molecules of Glc and one molecule of Ara [M-3Glc-Ara-H]^−^. The precursor ion was [M-H]^−^ at *m*/*z* 945.5 for Rd, and the product ion peak at *m*/*z* 621.5, which correspond to the loss of two molecules of Glc [M-2Glc-H]^−^. The precursor ion was [M-H]^−^ at *m*/*z* 945.5 for Re, and the product ion peak was at *m*/*z* 475.5, which correspond to the loss of two molecules of Glc and one molecule of Rha [M-2Glc-Rha-H]^−^. The precursor ion was [M-H]^−^ at *m*/*z* 867.6 for internal standard (IS), and the product ion peak was at *m*/*z* 721.0, which correspond to the loss of one molecule of Rha [M-Rha-H]^−^. Table 1 summarizes the main MS parameters, including cone voltage (CV) and collision energy (CE).

Under the above-mentioned chromatographic conditions, saponin components showed more intensive deprotoned ions in the negative ion mode than those of protonated or sodiated precursors in the positive mode. Therefore, an UPLC-MRM-MS with an ESI interface in the negative mode was used for separation and detection of all compounds tested. Reference standards showed good peak shape and excellent resolution. Figure 2 shows the typical mass spectra and MS/MS spectra of Rb_1_, Rb_2_, Rd, Re, and dioscin. Dioscin was used as the IS. The chemical structure of IS was found to be similar to analytes, and it exhibited a stable response, indicating effective separation.

### 3.2. Method Validation

#### 3.2.1. Selectivity

**Figure 3:**
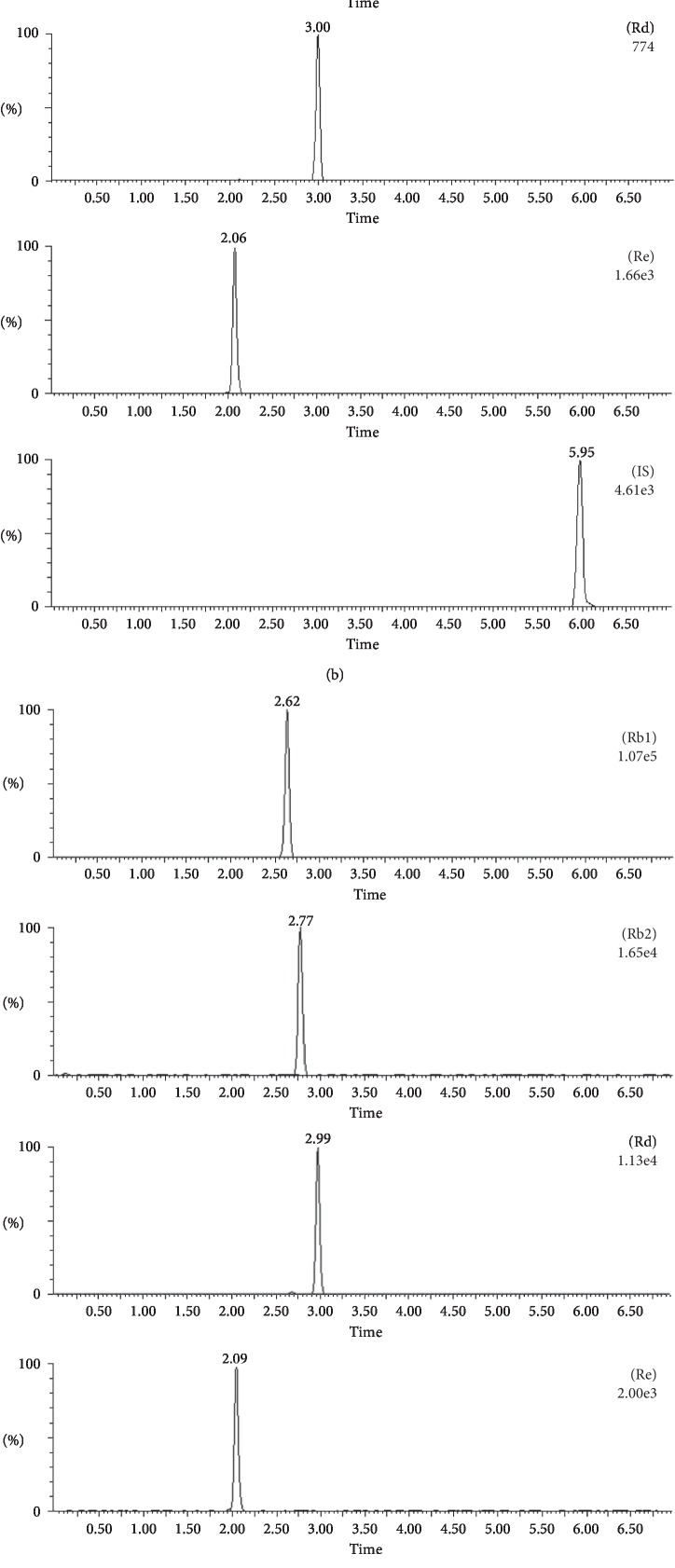
Representative MRM chromatograms of Rb_1_, Rd, Re, Rb_2_, and IS in rat plasma samples. Blank rat plasma (a), a blank plasma with IS and analytes at MQC levels (b), a plasma sample taken 8 h after oral administration of PQ (c), and PQ-AG extracts (d).

Figure 3 illustrates the typical multiple reaction monitoring (MRM) chromatograms from blank plasma sample and the sample at 8 h after oral administration of PQ and PQ-AG extracts. No interference peaks were detected during the retention time of IS as well as Rb_1_, Rb_2_, Rd, and Re.

#### 3.2.2. Linearity and Lower Limit of Quantification (LLOQ)

Table 2 displays the typical equations of calibration curves for the four analytes. The correlation coefficient (*r*) for each calibration curve was higher than 0.9978. The result indicated that the ratio of peak area strongly correlated with the concentration of each compound within the acceptable linearity ranges. The LLOQs of the four analytes are shown in Table 2.

#### 3.2.3. Precision and Accuracy

The precision and accuracy of the assay were validated for the samples at LLOQ and three QC levels analyzed on the same day or during three consecutive days. The results are presented in Table 3. The intraday precision and interday precision ranged from 4.57% to 14.63%.

#### 3.2.4. Extraction Recovery and ME

The recoveries were obtained at three concentration levels, ranging from 76.43% to 95.20%. The ME of all the analytes in blank plasma were within the acceptable range of 90.44%–107.11% (Table 4). The ME of IS was 92.30%. Therefore, it demonstrated that ME of the plasma was negligible in the assay.

#### 3.2.5. Stability

The sample stability during storage and processing procedures was evaluated by the analysis of QC samples (Table 5), showing that these analytes were stable after short-term storage (1-month storage at −80°C), postpreparative stability (12 h in the autosampler at 4°C), and three freeze-thaw cycles. All compounds remained stable for 2 weeks at −80°C.

### 3.3. Pharmacokinetics Study

**Figure 4:**
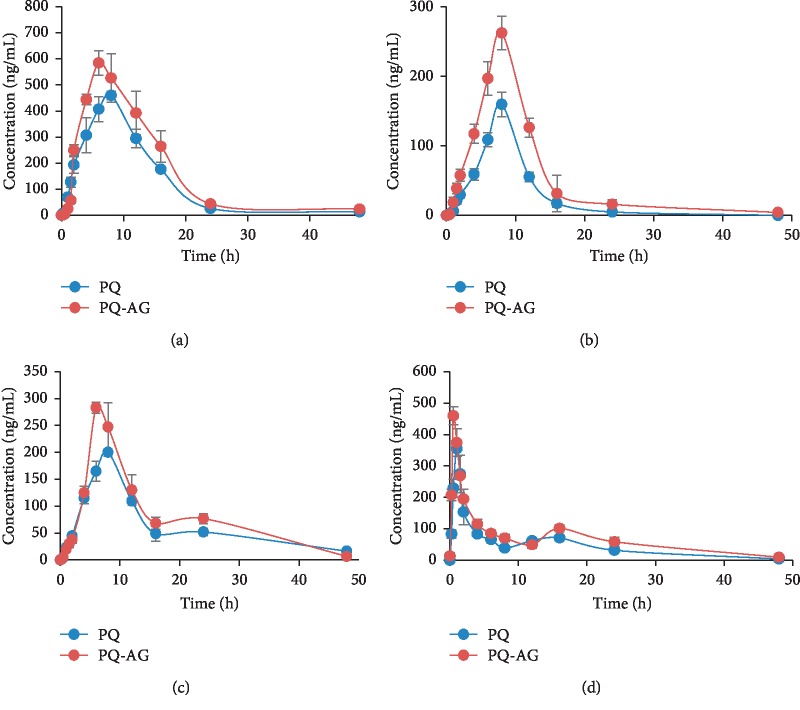
Mean concentration-time profiles of Rb_1_ (a), Rb_2_ (b), Rd (c), and Re (d) after oral administration of PQ-AG and PQ extracts (*n* = 6, mean ± SD).

Figure 4 shows the mean plasma concentration-time curves of Rb_1_, Rb_2_, Rd, and Re after oral administration of PQ-AG or PQ.  Table 6 summarizes the pharmacokinetic parameters of noncompartment model, including maximum plasma concentration (*C*_max_), time to reach the maximum concentrations (*T*_max_), half-time (*t*_1/2_), and area under concentration-time curve (AUC_0⟶*t*_ and AUC_0⟶∞_). These results showed that UPLC-MS/MS could be used to detect Rb_1_, Rb_2_, Rd, and Re, revealing good absorption of four compounds. The significant differences among Rb_1_, Rb_2_, Rd, and Re were observed between PQ-AG and PQ groups after oral administration. For Rb_1_ with certain bioavailability [20], the plasma *C*_max_ was 600.2 ± 53.6 ng/mL in the PQ-AG group, which was significantly higher than that (461.2 ± 15.9 ng/mL) of the PQ group (*P* < 0.05). Meanwhile, the *T*_max_ of Rb_1_ was 6.67 ± 1.03 h for the PQ-AG group and 7.00 ± 1.10 h for the PQ group, illustrating Rb_1_ with high uptake in rat plasma for the PQ-AG group. The reason might be attributed to those other components in the PQ-AG group which promoted the absorption and metabolism of Rb_1_. The *C*_max_ and *T*_max_ values of Rb_2_ in the PQ-AG group were 264.1 ± 24.5 ng/mL and 7.67 ± 0.82 h, respectively. The *C*_max_ and *T*_max_ values of Rb_2_ in the PQ group were 160.1 ± 18.8 ng/mL and 7.67 ± 0.82 h, respectively. Rd showed a good absorption in the PQ-AG group, indicating that *C*_max_ was 283.4 ± 11.1 ng/mL and *T*_max_ was lower than that in the PQ group (*P* < 0.05), in which the *C*_max_ was 200.9 ± 7.3 ng/mL and *T*_max_ was 7.67 ± 0.82 h. The results also showed that the *T*_max_ of Rd and Re in the PQ-AG group was shorter than those in the PQ group (*P* < 0.05). Moreover, as shown from Figure 4 and Table 6, the *T*_max_ value of Re, which was different from the other three compounds between the two groups, was 0.58 ± 0.20 and 1.08 ± 0.20 h. Although the entire hypothesis needed to be further validated, Rd and Re had different absorptions compared with Rb_1_ and Rb_2_, and the double-peak phenomenon was detected at the same time, which could be probably attributed to the enterohepatic circulation in drug metabolism or the interactivity of PQ-AG drug pair [21, 22]. In addition, *C*_max_, AUC_0⟶*t*_, and AUC_0⟶∞_ of the four active ingredients in the PQ-AG group were higher than those in the PQ group. It suggested that four compounds in the PQ-AG group had synergistic effects with other compounds, leading to increased plasma contents of four analytes by promoting absorption. Compared with the PQ group (*P* < 0.05), the *t*_1/2_ of four analytes in the PQ-AG group was 18.52 ± 2.61, 15.61 ± 5.21, 14.21 ± 1.98, and 9.81 ± 2.15 h, respectively, indicating slow elimination. Moreover, the absorption was enhanced after compatibility, and the duration of drug action was prolonged for *t*_1/2_ of the four compounds in the PQ-AG group. Furthermore, the bioavailability of bioactive components in the PQ-AG group showed an upward trend, indicating that the four bioactive compounds of Rb_1_, Rb_2_, Rd, and Re were well absorbed and gradually removed in the PQ-AG group, which might be attributed to the synergic action between PQ and AG. According to TCM theory, the compatibility mechanisms of drug pairs are not only arbitrary plus of two drugs but also the regularity of active components in vivo [23]. In the PQ-AG group, the dissolutions of various chemical constituents were increased due to the good absorption of ginsenosides in PQ [24]. We ventured to guess that the volatile components of AG probably enhanced the absorption of ginsenosides in PQ [25, 26]. In the PQ-AG group, the ginsenoside possessed a greater uptake and slower elimination, which was connected with the interaction between AG and PG, including the mediations of transport proteins, metabolic enzymes, and plasma protein binding. Definitely, the detailed mechanisms of PQ-AG drug pairs needed to be further investigated.

The results showed that AG enhanced the absorption and delayed the elimination of the four analytes in PQ. Moreover, it could result in a better bioavailability of ginsenosides. Some previous literatures have shown that certain compounds in AG may suppress P-glycoprotein functions in the intestinal membrane and the activity of enzyme CYP1A2 [27]. These findings could probably explain why the main components of PQ accumulated after PQ-AG was simultaneously administered. Although these hypotheses needed to be further investigated, we believed that AG and the compatibility of ingredients could result in a better absorption of ginsenosides in PQ.

## 4. Conclusions

In the present study, we successfully applied a simple, rapid, and sensitive UPLC-MS/MS method to simultaneously determine four bioactive ginsenosides in PQ and the combination of PQ and AG. We, for the first time, reported pharmacokinetic parameters of Rb_1_, Rb_2_, Rd, and Re in PQ-AG after oral administration. Our data showed that the four analytes had a better absorption and slower elimination after PQ and AG were combined. The changes of four main active substances in vivo provided valuable insights into the compatibility principles of PQ-AG. In addition, our current findings clarified the rational compatibility of TCM, suggesting better clinical application and research potentials.

## Figures and Tables

**Table 1:** Precursor/production pairs and parameters for MRM of ginsenosides.

Analytes	Ionization mode	[M-H]^−^(*m/z*)	MRM transitions (precursor-product)	Cone voltage (V)	Collision energy (eV)
Rb_1_	Negative	1107.5	1107.5 ⟶ 621.2	80	40
			1107.5 ⟶ 459.6^*∗*^		45

Rb_2_	Negative	1077.7	1077.7 ⟶ 621.5	80	50
			1077.7 ⟶ 459.5^*∗*^		60

Rd	Negative	945.5	945.5 ⟶ 621.5^*∗*^	85	50
			945.5 ⟶ 459.5		45

Re	Negative	945.5	945.5 ⟶ 637.5	80	45
			945.5 ⟶ 475.5^*∗*^		40

Dioscin	Negative	867.6	867.6 ⟶ 721.0	85	45

^*∗*^Quantitative ion.

**Table 2:** The regression equations, linear ranges, and LLOQs of the four compounds.

Compound	Linear regression equation	Linear range (ng/mL)	*r*	LLOQ (ng/mL)
Rb_1_	*Y* = 2.823 × 10^−3^*X* + 9.654 × 10^−2^	3.6–1139	0.9987	3.6
Rb_2_	*Y* = 7.729 × 10^−4^*X* + 2.824 × 10^−3^	1.6–500.0	0.9989	1.6
Rd	*Y* = 7.774 × 10^−3^*X* + 9.646 × 10^−2^	1.2–390.2	0.9979	1.2
Re	*Y* = 4.036 × 10^−4^*X* + 3.149 × 10^−3^	2.5–788.5	0.9978	2.5

**Table 3:** Precision and accuracy for the determination of the ginsenosides in rat plasma.

Compounds	Spiked concentration (ng/mL)	Measured (ng/mL)	Intraday precision RSD (%)	Interday precision RSD (%)	Accuracy Re (%)
Rb_1_	3.6	3.3 ± 0.21	12.01	13.73	3.40
	7.2	7.1 ± 0.10	7.55	7.02	−1.22
	142.4	143.1 ± 1.05	6.83	4.57	4.39
	911.4	910.9 ± 0.05	9.57	5.44	6.01

Rb_2_	1.6	1.7 ± 0.13	13.54	14.01	−1.84
	3.2	3.2 ± 0.05	14.32	14.48	−5.32
	62.5	61.5 ± 0.18	6.82	8.07	3.76
	400.0	399.3 ± 0.25	9.97	9.19	5.37

Rd	1.2	1.3 ± 0.17	14.61	10.22	4.20
	2.4	2.7 ± 0.21	13.57	13.74	−2.10
	48.8	49.5 ± 0.17	10.83	7.22	−4.05
	312.2	311.9 ± 0.26	6.39	6.04	5.01

Re	2.5	2.5 ± 0.07	11.65	10.34	−6.94
	5.0	4.89 ± 0.26	14.63	14.57	3.29
	98.6	98.3 ± 0.04	8.77	11.22	7.35
	630.8	632.8 ± 0.31	12.08	7.58	−4.10

**Table 4:** Recoveries and matrix effects of the ginsenosides in rat plasma.

Compound	Concentration (ng/mL)	Extraction recovery		Matrix effect	
		Mean (%)	RSD (%)	Mean (%)	RSD (%)
Rb_1_	7.2	77.51	6.31	93.37	8.12
	142.4	85.73	1.52	98.36	4.33
	911.4	76.43	13.15	90.44	8.79

Rb_2_	3.2	79.62	2.50	91.11	8.01
	62.5	86.89	10.74	97.83	7.52
	400.0	78.15	4.62	102.10	3.77

Rd	2.4	86.64	13.60	99.67	12.74
	48.8	95.20	10.17	100.29	8.88
	312.2	88.58	6.51	106.2	13.74

Re	5.0	82.34	6.74	102.56	11.12
	98.6	86.32	3.33	99.52	7.65
	630.8	85.79	12.10	107.11	15.02

IS	720	89.31	5.76	92.30	6.47

**Table 5:** Stability of the ginsenosides in rat plasma.

Compound	Spiked concentration (ng/mL)	Mean concentration (ng/mL)	Re (%)			Recovery (%)		
			Short term	Freeze-thaw cycles	Postpreparation	Short term	Freeze-thaw cycles	Postpreparation
Rb_1_	7.2	7.1	−7.55	−6.90	6.86	96.32	97.66	101.85
	142.4	141.2	7.95	−4.48	9.35	100.48	95.35	101.65
	911.4	910.5	7.53	7.75	−6.47	101.65	101.95	96.10

Rb_2_	3.2	3.0	−9.70	−9.69	−9.72	93.67	94.21	93.37
	62.5	61.6	−2.12	6.77	2.39	95.33	100.41	99.94
	400.0	402.9	7.11	9.36	7.90	98.74	103.82	99.63

Rd	2.4	2.4	8.64	−11.93	4.43	102.66	97.24	100.10
	48.8	49.7	10.24	8.87	9.98	103.61	100.20	101.71
	312.2	310.2	7.35	8.12	−7.02	101.27	102.00	94.81

Re	5.0	5.1	−10.71	7.33	8.54	97.30	104.07	104.63
	98.6	95.5	12.11	7.53	8.92	99.25	94.12	97.21
	630.8	634.2	8.40	−9.85	9.76	101.73	97.39	102.50

**Table 6:** Pharmacokinetic parameters of the ginsenosides after oral administration of PQ-AG and PQ extracts in rats (*n* = 6, mean ± SD).

Group	Compounds	*C*_max_ (ng/mL)	*T*_max_ (h)	*t*_1/2_ (h)	AUC_0⟶*t*_ (ng·h/mL)	AUC_0⟶∞_ (ng·h/mL)
PQ-AG	Rb_1_	600.2 ± 53.6^*∗*^	6.67 ± 1.03	18.52 ± 2.61^*∗*^	8132.09 ± 454.40^*∗*^	8414.43 ± 487.69^*∗*^
	Rb_2_	264.1 ± 24.5^*∗*^	7.67 ± 0.82	15.61 ± 5.21^*∗*^	2458.74 ± 242.86^*∗*^	2518.20 ± 288.99^*∗*^
	Rd	283.4 ± 11.1^*∗*^	6.33 ± 0.82^*∗*^	14.21 ± 1.98^*∗*^	3874.28 ± 356.39^*∗*^	3955.23 ± 382.94^*∗*^
	Re	460.4 ± 30.2^*∗*^	0.58 ± 0.20^*∗*^	9.81 ± 2.15^*∗*^	3427.96 ± 293.76^*∗*^	3582.89 ± 396.42^*∗*^

PQ	Rb_1_	461.2 ± 15.9	7.00 ± 1.10	15.61 ± 1.52	5983.67 ± 313.47	6146.74 ± 328.31
	Rb_2_	160.1 ± 18.8	7.67 ± 0.82	13.34 ± 1.58	1214.41 ± 99.25	1229.91 ± 99.58
	Rd	200.9 ± 7.3	7.67 ± 0.82	13.11 ± 2.61	3005.92 ± 229.63	3324.67 ± 317.05
	Re	354.7 ± 26.6	1.08 ± 0.20	8.14 ± 1.20	2331.52 ± 214.10	2379.76 ± 223.68

^*∗*^ *P* < 0.05 compared with PQ group.

## Data Availability

All data contained in the manuscript will be made available from the corresponding author upon reasonable request.
